# ALFY localizes to early endosomes and cellular protrusions to facilitate directional cell migration

**DOI:** 10.1242/jcs.259138

**Published:** 2022-02-18

**Authors:** Kristiane Søreng, Serhiy Pankiv, Camilla Bergsmark, Ellen M. Haugsten, Anette K. Dahl, Laura R. de la Ballina, Ai Yamamoto, Alf H. Lystad, Anne Simonsen

**Affiliations:** 1 Department of Molecular Medicine, Institute of Basic Medical Sciences, University of Oslo, 0317 Oslo, Norway; 2 Centre for Cancer Cell Reprogramming, Institute of Clinical Medicine, University of Oslo, 0318 Oslo, Norway; 3 Department of Tumor Biology, Institute for Cancer Research, Oslo University Hospital, Montebello, 0379 Oslo, Norway; 4 Department of Neurology, Pathology and Cell Biology, Columbia University, New York, NY 10032-3784, USA; 5 Department of Molecular Cell Biology, Institute for Cancer Research, Oslo University Hospital, Montebello, 0379 Oslo, Norway

**Keywords:** ALFY, WDFY3, Endosome, Migration, Focal adhesion, Integrin

## Abstract

Cell migration is a complex process underlying physiological and pathological processes such as brain development and cancer metastasis. The autophagy-linked FYVE protein (ALFY; also known as WDFY3), an autophagy adaptor protein known to promote clearance of protein aggregates, has been implicated in brain development and neural migration during cerebral cortical neurogenesis in mice. However, a specific role of ALFY in cell motility and extracellular matrix adhesion during migration has not been investigated. Here, we reveal a novel role for ALFY in the endocytic pathway and in cell migration. We show that ALFY localizes to RAB5- and EEA1-positive early endosomes in a PtdIns(3)P-dependent manner and is highly enriched in cellular protrusions at the leading and lagging edge of migrating cells. We find that cells lacking ALFY have reduced attachment and altered protein levels and glycosylation of integrins, resulting in the inability to form a proper leading edge and loss of directional cell motility.

## Abstract (precis)

**Summary:** The autophagy-linked FYVE protein (ALFY) localizes to cell protrusions and early endosomes, and regulates directional cell migration and attachment by modification of integrin glycosylation.

## INTRODUCTION

ALFY (also known as WDFY3) is a large evolutionarily conserved protein known to promote clearance of protein aggregates through its interaction with autophagy components and phosphatidylinositol-3-phosphate [PtdIns(3)P] (Simonsen et al., 2004; Lystad et al., 2014; Clausen et al., 2010; Filimonenko et al., 2010; Han et al., 2015; Fox et al., 2020). Depletion of ALFY leads to accumulation of ubiquitin-positive aggregates (Filimonenko et al., 2010; Finley et al., 2003), and its heterozygous depletion significantly accelerates age of onset and pathogenesis in a mouse model of Huntington's disease (Fox et al., 2020; Dragich et al., 2016). ALFY knockout (KO) mice die a few hours after birth and are characterized by loss and disorganization of interhemispheric axonal tracts throughout the brain, suggesting that ALFY is involved in neural migration during cerebral cortical neurogenesis (Dragich et al., 2016; Kadir et al., 2016; Orosco et al., 2014).

Cell migration involves cell polarization to develop a leading and a trailing end, followed by recruitment of the actin cytoskeleton and formation of cell protrusions and focal adhesions (FAs) at the leading edge. FAs are large complexes consisting of multiple scaffolding and signaling proteins, such as integrins, paxillin and vinculin, which link the extracellular matrix to intracellular signaling pathways, leading to actin remodeling and traction required for forward cellular movement (Winograd-Katz et al., 2014). Dynamic assembly and disassembly of FAs is important for productive displacement of the cell body as the cell moves (Kenific and Debnath, 2016). During cell migration, integrins, the transmembrane proteins of FAs, are internalized into RAB5-positive early endosomes, from where they can be targeted for lysosomal degradation or recycled to the plasma membrane of the leading edge, via RAB11-positive recycling endosomes (note mammals have more than one RAB5, RAB7 and RAB11, and here we refer to them generically unless otherwise specified), to facilitate another round of cell motility (Maritzen et al., 2015; Shafaq-Zadah et al., 2016; Nader et al., 2016).

Here, we report a novel role for ALFY in directional cell migration. We show that ALFY is highly enriched in cellular protrusions and early endosomes, and that it colocalizes with integrin-α5 and regulates protein levels and glycosylation of several integrins, implying it regulates integrin trafficking during directional cell migration.

## RESULTS AND DISCUSSION

### ALFY localizes to cellular protrusions

**Fig. 1.**
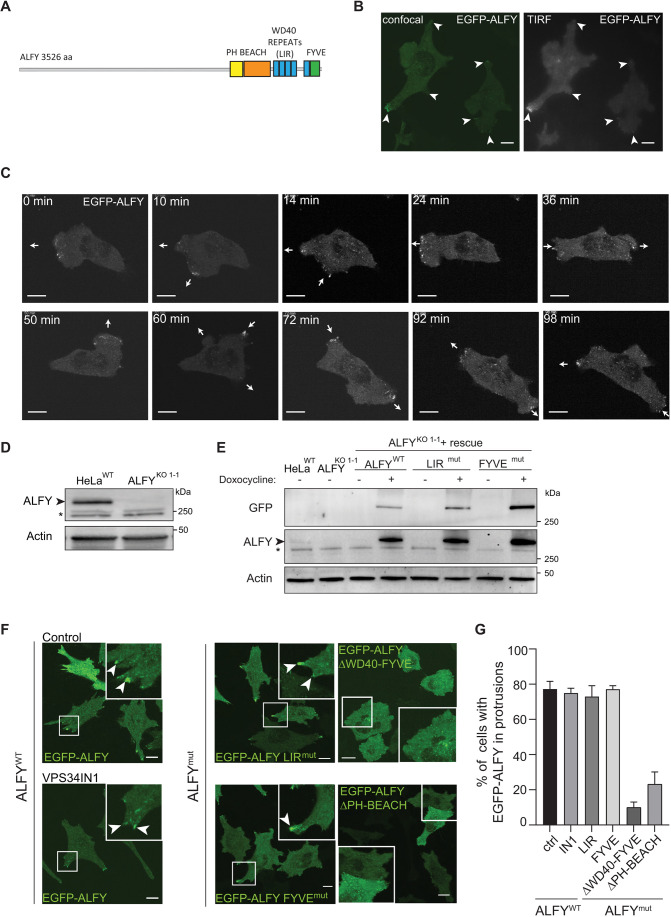
**ALFY localizes to cellular protrusions.** (A) Domain structure of ALFY. ALFY contains a long N-terminus, followed by a conserved C-terminus with a PH-BEACH-domain, five WD repeats including an LC3-interacting region (LIR) and a PtdIns(3)P-binding FYVE-domain. (B) HeLa cells with inducible expression of EGFP–ALFY were treated with tetracycline for 24 h and imaged live with spinning disc confocal (left) or TIRF (right) imaging modes. Arrowheads highlight accumulated fluorescence signal in cellular protrusions. Scale bars: 10 µm. (C) HeLa^KO1-1^ cells stably transfected with 3×Flag–EGFP–ALFY were imaged live for 100 min. Arrows illustrate the movement direction of leading and lagging ends of the cell. Scale bars: 10 µm. (D) Western blot of HeLa T-Rex FlpIN^WT^ and ALFY^KO1-1^ cell lines generated by CRISPR-Cas9. *indicates unspecific protein bands. (E) Western blot of HeLa T-Rex FlpIN^WT^, ALFY^KO1-1^ and ALFY^KO1-1^ rescue cells treated or not with doxycycline to induce expression of EGFP-ALFY, LIR^mut^ and FYVE^mut^. * indicates unspecific bands. Images in B–E are representative of three experiments. (F) ALFY^KO1-1^ rescue cell lines were treated with doxycycline for 24 h to induce expression of EGFP–ALFY, EGFP–ALFY LIR^mut^, EGFP–ALFY FYVE^mut^, EGFP–ALFY ΔWD40-FYVE and EGFP–ALFY ΔPH-BEACH followed by live-cell imaging. EGFP-ALFY^WT^ cells were treated or not with VPS34IN1 for 2 h (left panel) prior to imaging. Arrowheads highlight accumulated fluorescence signal in cellular protrusions. Scale bars: 10 µm. (G) Quantification of the percentage (%) of cells with EGFP–ALFY in protrusions from the images in F (mean±s.e.m. from 35–100 cells per cell line).

ALFY contains several C-terminal domains, including a PH-BEACH-domain with unknown function, five WD repeats, an LC3-interacting region (LIR) that binds to GABARAP proteins and a PtdIns(3)P-binding FYVE-domain (Simonsen et al., 2004; Lystad et al., 2014) (Fig. 1A). The large size of ALFY (3526 amino acids, 68 exons) has made it difficult to clone and express the full-length protein and previous studies have therefore been based on immunostaining of fixed cells with an anti-ALFY antibody or expression of deletion mutants (Clausen et al., 2010; Simonsen et al., 2004). We have now successfully cloned and expressed full-length ALFY, tagged with enhanced green fluorescent protein (EGFP) or tandem-dimer NeonGreen (tdNG), in HeLa and U2OS T-Rex Flp-In cells, respectively (Fig. S1A,B)*.* Live-cell imaging of both cell lines shows ALFY localization to small dynamic vesicle-like structures distributed throughout the cytoplasm, with enrichment in cellular protrusions and close to the basal plasma membrane (Fig. 1B; Fig. S1C). Interestingly, the ALFY-positive structures were actively redistributed to the advancing leading edge and retracting lagging edge of migrating cells (Fig. 1C), indicating a role for ALFY during cell migration.

To further study the localization and function of wild-type (WT) and mutant ALFY proteins, ALFY was depleted in HeLa T-Rex Flp-In cells (HeLa ALFY^KO^) with two different guide RNAs (Fig. S1D), as confirmed by western blotting (Fig. 1D; Fig. S1E) and immunoprecipitation with several anti-ALFY antibodies (Fig. S1F). Several KO clones were selected for further studies (ALFY^KO1-1^, ALFY^KO2-6^, ALFY^KO2-9^ and ALFY^KO2-11^) and for generation of rescue cell lines with inducible expression of EGFP–ALFY WT, LIR or FYVE domain mutants (Figs 1E and 4B). Analogous to what was seen for EGFP–ALFY, both EGFP–ALFY LIR^mut^ and FYVE^mut^ localized to intracellular structures and to cellular protrusions (Fig. 1F,G), indicating that localization of ALFY to protrusions occurs independently of its binding to GABARAP proteins and PtdIn(3)P, respectively (Simonsen et al., 2004; Lystad et al., 2014). Likewise, localization of EGFP–ALFY to protrusions was unaffected in cells treated with the VPS34 inhibitor (VPS34IN1) (Fig. 1F,G). In contrast, ALFY mutants lacking either the WD40-FYVE domains (EGFP–ALFY ΔWD40-FYVE) or the PH-BEACH domains (EGFP–ALFY ΔPH-BEACH) did not show an enrichment in cellular protrusions (Fig. 1F,G), suggesting a role for its C-terminal part in targeting to cellular protrusions.

### ALFY localizes to early endosomes in a PtdIns(3)P-dependent manner

**Fig. 2.**
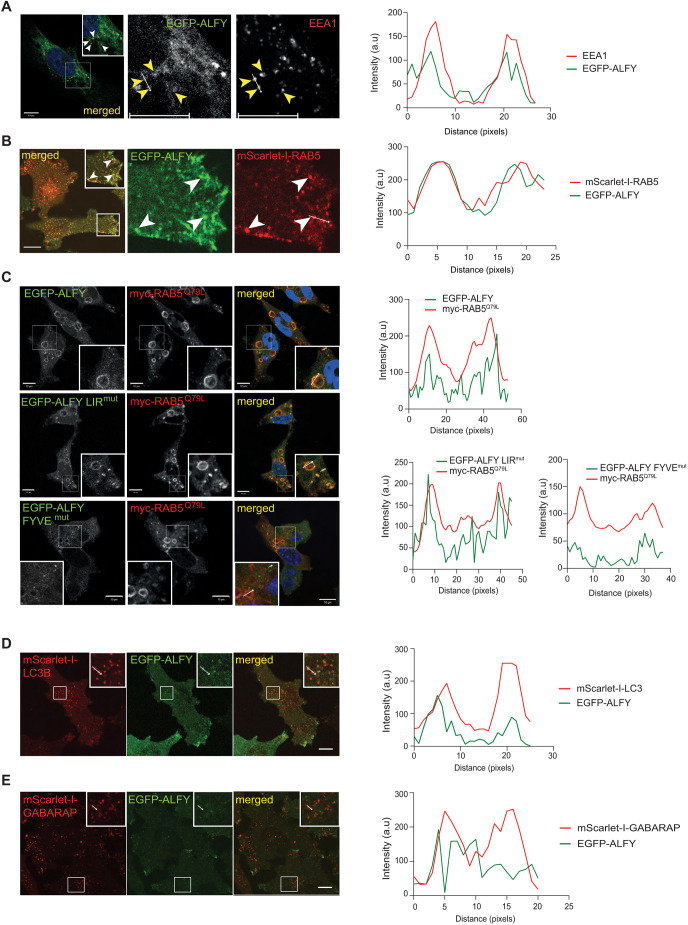
**ALFY localizes to early endosomes in a PtdIns(3)P-dependent manner.** (A) ALFY^KO1-1^ EGFP–ALFY rescue cells were fixed and immunostained against EEA1 and analyzed by confocal microscopy. Yellow (white in inset) arrowheads highlight EGFP–ALFY structures positive for EEA1. The colocalization histogram is from the two vesicles in the line marked. (B) ALFY^KO1-1^ EGFP–ALFY cells stably expressing mScarlet-I–RAB5 were treated with tetracycline overnight and analyzed with live-cell imaging. mScarlet-I–RAB5- and EGFP–ALFY-positive puncta are indicated by arrowheads, and colocalization is shown in the histogram for the line marked. (C) HeLa ALFY^KO1-1^ cells rescued with EGFP–ALFY, EGFP–ALFY LIR^mut^ or EGFP–ALFY FYVE^mut^ were transiently transfected with Myc–RAB5^Q79L^, fixed at 18 h post transfection, and immunostained against Myc for visualization of the RAB5^Q79L^ structures before confocal microscopy analysis. Colocalization histograms from the indicated Myc–RAB5^Q79L^ structures are shown. (D) ALFY^KO1-1^ EGFP–ALFY cells with stable expression of mScarlet-I-tagged LC3B were treated with tetracycline and imaged live. Colocalization was measured from the indicated structures. (E) ALFY^KO1-1^ EGFP–ALFY cells with stable expression of mScarlet-I-tagged GABARAP were treated with tetracycline and imaged live. Colocalization was measured from the indicated structures. Images are representative of three experiments. Scale bars: 10 µm.

The nature of the EGFP–ALFY-positive structures was analyzed by co-immunostaining for different cytoplasmic markers. Surprisingly, EGFP–ALFY colocalized extensively with the early endosome markers EEA1 and RAB5 (Fig. 2A,B; Movie 1), while dynamic ‘kiss and run’ interactions were detected with the late endosome marker RAB7 or the recycling endosome marker RAB11 (Fig. S1G,H, Movies 2, 3). We noticed during live-cell imaging that the subcellular localization of EGFP–ALFY is highly sensitivity to temperature, as its localization to cellular protrusions was lost upon washing cells with cold PBS for 10 min at room temperature (Fig. S1I). Given that many intracellular events, including endocytosis and secretion, are inhibited at low temperature (Saraste et al., 1986; Tomoda et al., 1989), and the weak binding of the ALFY FYVE domain to PtdIns(3)P-containing membranes (Reinhart et al., 2021), it is likely that recruitment of ALFY to early endosomes might be activity dependent.

As neither the LIR nor the FYVE domain of ALFY are essential for its localization to protrusions (Fig. 1F,G), we asked whether these motifs are required for recruitment of ALFY to early endosomes. HeLa ALFY^KO1-1^ cells expressing EGFP–ALFY, EGFP–ALFY LIR^mut^ or EGFP–ALFY FYVE^mut^ were transfected with a constitutively active GTPase-deficient RAB5 mutant (Myc–RAB5^Q79L^) that generates enlarged early endosomes. Both EGFP–ALFY and EGFP–ALFY LIR^mut^ were efficiently recruited to the Myc–RAB5^Q79L^ vesicles, while recruitment of EGFP–ALFY FYVE^mut^ was strongly reduced (Fig. 2C), indicating that recruitment of ALFY to early endosomes requires its FYVE domain, but is independent of its binding to GABARAP . The EGFP–ALFY FYVE^mut^ did however localize to small RAB5-negative structures (Fig. 2C; Fig. S2B), in line with the presence of EGFP–ALFY-positive structures in cells treated with VPS34IN1 (Fig. 1F).

As reported previously (Simonsen et al., 2004; Lystad et al., 2014), we observed some colocalization of EGFP–ALFY with the autophagy markers mScarlet-I–LC3B (LC3B is also known as MAP1LC3B) (Fig. 2D) and mScarlet-I–GABARAP (Fig. 2E), while full-length ALFY failed to colocalize with the autophagy receptor mScarlet-I–SQSTM1 under basal conditions (Fig. S2A).

To elucidate a possible endosomal function of ALFY, we analyzed recycling of transferrin (Tfn) and degradation of the Tfn receptor (TfR) as well as degradation of epidermal growth factor (EGF)-receptor (EGFR) and Rhodamine–EGF in WT and ALFY^KO1-1^ cells. Whereas Tfn recycling and TfR degradation was unaffected in ALFY^KO1-1^ cells (Fig. S2C–E), the level of EGFR and Rhodamine–EGF was reduced in ALFY^KO1-1^ cells compared to that in WT cells (Fig. S2F–J), suggesting that ALFY regulates cargo sorting of EGFR from early endosomes to lysosomes.

Taken together, we show that EGFP–ALFY localizes to early endosomes in a PtdIns(3)P-dependent manner, but that it also is recruited to cytoplasmic structures independently of PtdIns(3)P.

### ALFY is required for directional cell migration

**Fig. 3.**
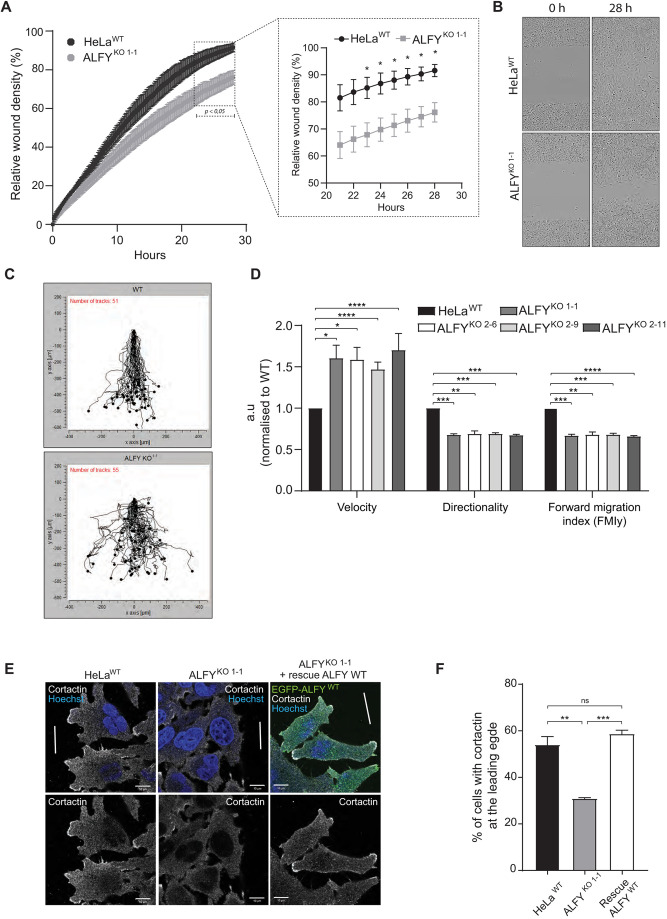
**ALFY is required for directional cell migration.** (A) Wound healing analysis of HeLa^WT^ and ALFY^KO1-1^ cells using the Incucyte^®^ live-cell imaging system. The mean relative wound density was quantified from three replicates from three independent experiments. The timepoints marked by a dotted square in the left panel are shown as a magnified graph to the right (mean±s.e.m., *n*=3). *P<0.05 by multiple unpaired two-tailed *t*-tests. (B) Representative images from experiments as in A showing the wound densities of HeLa^WT^ and ALFY^KO1-1^ at 0 and 28 h. Images show 1460×1970 μm. (C) Graphs representing movements of HeLa^WT^ and ALFY^KO1-1^ cells obtained by tracking individual cells in the images from experiments as in A using the Chemotaxis and migration tool (Ibidi). (D) Manual tracking of images from wound healing assays of HeLa^WT^, ALFY^KO1-1^, ALFY^KO2-6^, ALFY^KO2-9^ and ALFY^KO2-11^, showing velocity, directionality and forward migration index along the *y*-axis (mean±s.e.m., *n*=3). *P<0.05, **P<0.01; ***P<0.001; ****P<0.0001 (unpaired two-tailed Student's *t*-test). (E) Analysis of leading edge formation in HeLa^WT^ cells, ALFY^KO1-1^ cells and ALFY^KO1-1^ rescue cells with doxycycline-induced expression of EGFP–ALFY, immunostained against cortactin for marking the leading edge. The white lines indicate the introduced wound. (F) Quantification of the percentage (%) of cells from E with cortactin at the leading edge. (mean±s.e.m., *n*=3). **P<0.01; ***P<0.001; ns, not significant (unpaired two-tailed Student's *t*-test).

The localization of ALFY to the leading and lagging edges of migrating cells, as well as its role in neural migration during cerebral cortical neurogenesis, prompted us to investigate a role for ALFY in cell migration. Wound healing analysis for 28 h revealed that ALFY^KO^ cells showed reduced wound closure (Fig. 3A,B), but similar proliferation compared to HeLa^WT^ cells (Fig. S3A), indicating that ALFY^KO^ cells have a diminished ability to migrate. By manually tracking individual cells, we found that ALFY^KO^ cells migrate faster than HeLa^WT^ cells, but lack directionality, and thereby fail to migrate into the wound (*y*-direction, FMIy) (Fig. 3C,D; Fig. S3B). This is reminiscent of the axon pathfinding defects observed in the ALFY KO mice, where axons failed to respond to chemotaxic cues (Dragich et al., 2016).

Moreover, recruitment of cortactin, an actin branching-promoting protein that is recruited to the leading edge of cells during cell migration (Ammer and Weed, 2008), was significantly abrogated in ALFY^KO1-1^ cells compared to HeLa^WT^ cells, but rescued by expression of EGFP–ALFY (Fig. 3E,F), indicating that ALFY promotes formation of a leading edge during directional cell migration, in line with its enrichment in cell protrusions at the leading edge (Fig. 1C) and reduced directionality of ALFY^KO^ cells (Fig. 3C,D; Fig. S3B).

### ALFY regulates cell attachment and integrin glycosylation

**Fig. 4.**
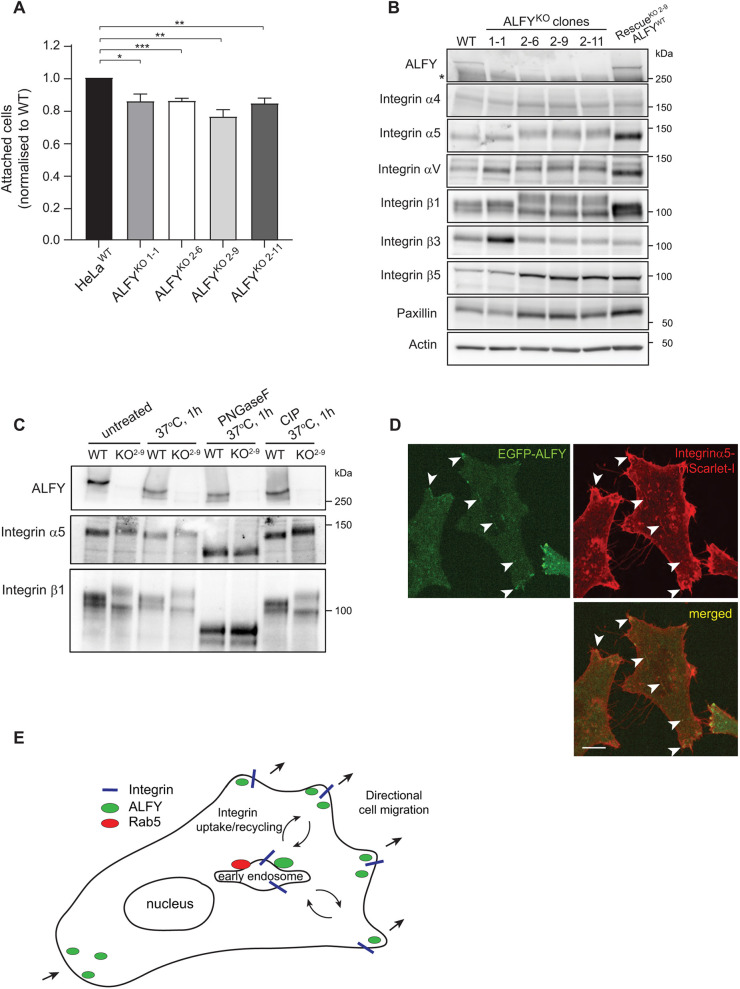
**ALFY regulates cell attachment and integrin trafficking.** (A) Quantification of proportion of attached HeLa^WT^ and ALFY^KO1-1^, ALFY^KO2-6^, ALFY^KO2-9^ and ALFY^KO2-11^ cells relative to wild type (stained with CellMask) (mean±s.e.m., *n*=4). *P<0.05; **P<0.01; ***P<0.001 (unpaired two-tailed Student's *t*-test). (B) Analysis of integrin protein levels from HeLa^WT^ and ALFY^KO1-1^, ALFY^KO2-6^, ALFY^KO2-9^ and ALFY^KO2-11^ cells as well as ALFY^KO2-9^ EGFP–ALFY rescue cells. The blots are representative of three independent experiments. (C) HeLa^WT^ and ALFY^KO2-9^ were treated or not with PNGaseF or CIP for 1 h at 37°C, followed by western blot analysis using the indicated antibodies. (D) Representative live-cell image of ALFY^KO2-9^ EGFP–ALFY rescue cells, co-expressed with integrin-α5–mScarlet-I. Arrowheads indicate structures positive for both. Scale bar: 10 µm. Images in C,D are representative of three experiments. (E) Model for role of ALFY in cell migration. ALFY localizes to cell protrusions in the leading and trailing edge of migrating cells, and to early endosomes. By being present on these structures, ALFY regulates the proper sorting and bi-directional trafficking of adhesion proteins such as integrins, thereby controlling directional cell migration.

As ALFY localizes to cellular protrusions and regulates cell migration, we asked if ALFY might regulate turnover of FAs, being important for cells to attach and detach during cell migration. Indeed, there was a significant reduction in the attachment of ALFY^KO^ cells compared to HeLa^WT^ cells (Fig. 4A). To determine whether this is caused by changes in FA proteins, we examined the expression levels of integrin proteins in ALFY^KO^ and rescue cells. Interestingly, migration of the protein bands of integrin-α5, integrin-αV and integrin-β1 was reduced in the ALFY^KO2^ clones and this was rescued upon re-expression of EGFP–ALFY (Fig. 4B). Moreover, increased protein levels of integrin-αV and integrin-β3, as well as reduced levels of paxillin, were detected in the ALFY^KO1-1^ cell line (Fig. 4B). It is not clear why the two ALFY^KO^ clones affect different integrins, but as ALFY is a very large protein with several transcripts, and we cannot rule out that the two guide (g)RNAs (targeting different exons) result in expression of a part of ALFY that potentially could have dominant-negative functions, although no truncated proteins were observed with the available antibodies (Fig. S1E,F). However, the changes in integrins observed upon ALFY depletion could be rescued by expression of full-length ALFY, indicating that ALFY regulates trafficking and possibly post-translational modifications of integrins. To address the latter, WT and ALFY^KO^ cells were treated with PNGaseF enzyme to cleave N-glycans or calf intestinal phosphatase (CIP) to remove phosphate. The mobility shift difference of integrin-α5 and integrin-β1 in ALFY^KO^ cells was completely lost in cells treated with PNGaseF, whereas CIP treatment had no effect (Fig. 4C), demonstrating that ALFY regulates N-glycosylation of integrins. Both integrin-α5 and integrin-β1 contain several potential N-linked glycosylation sites, and their glycosylation can affect the heterodimerization and binding properties, cell migration and adhesion (Isaji et al., 2009, 2006; Marsico et al., 2018; Hang et al., 2017). The mechanism of how ALFY regulates glycosylation of integrins is unclear, but its colocalization with integrin-α5–mScarlet-I in cell protrusions and in intracellular structures (Fig. 4D), as well as with RAB5A and EEA1 (Fig. 2A,B; Movie 1), suggest that it might regulate endocytosis of integrins from the plasma membrane and their transport to the recycling compartments. We observed close apposition and short-term colocalization of ALFY with the recycling endosome marker RAB11A and the late endosome marker RAB7A (Fig. S1G,H, Movies 2, 3), supporting this hypothesis. Glycosylation of integrins in RAB11-positive recycling endosomes (Kitano et al., 2021) and trans-Golgi network or perinuclear recycling compartments, where integrins transverse during long-loop recycling (De Franceschi et al., 2015), can explain the different glycosylation pattern of integrin-α5β1 in ALFY^KO^ cells. Interestingly, N-glycosylation of integrin-α5 has been reported to regulate EGFR activation (Hang et al., 2015), which may explain the effect of ALFY^KO^ on EGFR turnover (Fig. S2F–J).

Live-cell imaging revealed enrichment of ALFY in highly mobile structures in close proximity to paxillin-decorated FAs, suggesting they can deliver or remove components of FAs (Fig. S3C). It is established that migrating cells must remove their assembled adhesions at the rear of the cell and re-introduce them at the leading edge of the cell to migrate forward (Bretscher, 1989, 1992). As we observed EGFP–ALFY-positive structures both in the leading and trailing edge of cells (Fig. 1C), we speculate that ALFY is involved in the bi-directional trafficking to and from early endosomes (Fig. 4E). Further experiments are, however, required to determine the nature of the ALFY-mediated trafficking and its role in glycosylation of integrins.

In this study, we report for the first time the intracellular localization and dynamics of full-length ALFY in live cells. We show that ALFY localizes to cell protrusions and early endosomes, from where it seems to regulate proper sorting of integrins, thereby controlling directional cell migration and attachment (Fig. 4E). Our results thus provide an explanation for the defective neuronal migration and pathfinding phenotypes observed in mice lacking Alfy (Dragich et al., 2016).

## MATERIALS AND METHODS

### Cell lines, media and inhibitors

The HeLa T-Rex Flp-In cell line (Tighe et al., 2008) was obtained as a kind gift from Anthony Thige and Stephen S. Taylor, University of Manchester, UK and U2OS T-Rex Flp-In cell line (Malecki et al., 2006) was a kind gift of Stephen C. Blacklow, Harvard Medical School, USA. The cells were maintained in Dubecco's modified Eagle's medium (DMEM; Gibco) supplemented with 10% fetal bovine serum (FBS), 5 U/ml penicillin and 50 µg/ml streptomycin, as well as 5 µg/ml blasticidin (Invitrogen) and 100 µg/ml zeocin (Invitrogen) to maintain the TET-repressor and the Flp-In site. The cell lines with a stably integrated gene in the Flp-In site, were kept in 5 µg/ml blasticidin and 100 µg/ml hygromycin B (VWR), and gene expression induced by a 17–24 h treatment with 1 µg/ml doxycycline (Clonetech/AH) or tetracycline (Sigma, T7660). Puromycin (Sigma) was used at 2 µg/ml for selection of cells transfected with the CRISPR plasmid. VPS34IN1 (Selleckchem) was used at 5 µg/ml for 2 h. Bafilomycin A1 (BafA1; Enzo, BML-CM110-0100) was used at 100 nM for inhibition of lysosomal degradation. All cell lines were routinely tested for contamination.

### Antibodies

The following primary antibodies were used for immunofluorescence and/or immunoblotting: rabbit anti-ALFY (1:1000; Novus Biologicals, NBP1-03332, Bethyl Laboratories, A301-869A, Abcam, ab84888, LSBio, Cat. No LS-C483076 and Simonsen et al., 2004) anti-EEA1 (1:250, BD Biosciences, 610457), anti-Myc (1:500, Abcam, ab9132), anti-cortactin (1:200, Millipore upstate, 05-180), anti-paxillin (1:1000, Abcam, ab32084), anti-β-actin (1:1000, Cell Signaling Technology, #3700), anti-Flag (1:1000, Sigma, F1804), anti-GFP (1:1000, Clontech, #632381), anti-integrin-α4 (1:1000, Cell Signaling Technology, 8440T), anti-integrin-α5 (1:1000, Cell Signaling Technology, 4705T), anti-integrin-αV (1:1000, Cell Signaling Technology, 4711T), anti-integrin-β1 (1:1000, Cell Signaling Technology, 9699T), anti-integrin-β3 (1:1000, Cell Signaling Technology, 13166T), anti-integrin-β5 (1:1000, Cell Signaling Technology, 3629T), anti-EGFR (1:5000, Fitzgerald, 20-ES04), anti-transferrin receptor (1:5000, Zymed, 13-6890), anti-mouse-IgG (Starbright 700, BioRad, 12004158), anti-rabbit-IgG (DyLight 800, Thermo Fisher Scientific, SA5-10044), anti-sheep-IgG (DyLight 594, Thermo Fisher Scientific, SA510056), anti-rabbit-IgG (HRP, Jackson, ImmunoResearch 111-035-144), anti-mouse-IgG (Cy3, Jackson, ImmunoResearch 715-165-151), anti-rabbit (DyLight 649, Jackson, ImmunoResearch 711-495-152), anti-mouse-IgG (AlexaFluor647, Invitrogen, A31571) and anti-goat-IgG (Cy5, Jackson, ImmunoResearch 705-175-147). DNA staining was performed with Hoechst 33342 (Invitrogen, H1399).

### Plasmids and cloning

ALFY was amplified by PCR from a human cDNA library in four fragments (base pairs 1–3028, 2256–5425, 5123–7570 and 7355–10581), and cloned into the PCR blunt II TOPO vector (Invitrogen). For generation of LIR and FYVE domain mutants, mutagenesis was performed using QuikChange Lightning Multi Site-Directed mutagenesis kit (Agilent) on the constructs containing the target ALFY sequence. ALFY fragments, with or without LIR or FYVE mutations, were joined by Gibson assembly in pENTR1A (Invitrogen) to generate full-length ALFY. The resulting full-length ALFY insert was verified by sequencing. ALFY constructs lacking PH-BEACH or WD40-FYVE domains were generated by restriction enzyme cloning from the full-length pENTR-ALFY. pDest-FlpIn-tdNGFlag-ALFY and pDest-FlpIn-EGFP-ALFY were generated using Gateway recombination cloning (Invitrogen) from pENTR1A-ALFY WT, LIR or FYVE mutants or ΔPH-BEACH- or ΔWD40-FYVE mutants. Vectors for stable transfection of mScarlet-I–RAB5A, mScarlet-I–RAB7A, mScarlet-I–Rab11A, mScarlet-I–LC3B, mScarlet-I–GABARAP and mScarlet-I–SQSTM1 were generated by restriction enzyme subcloning of corresponding cDNA into pLVX-puro lentiviral backbone (Takara Bio). To generate the Gateway destination vector expressing tandem dimer NeonGreen (pDestFlpIn-tdNGFlag), the cDNA encoding two copies of NeonGreen with 3×Flag tag as a linker sequence was synthesized *de novo* (ThermoFisher GeneArt Gene Synthesis) and clone instead of HA tag into pDestFlpInHA vector. pLVX-paxillin--mScarlet-I and pLVX-Integrin α5-mScarlet-I were generated by Gibson assembly. All constructs were verified by Sanger sequencing. pCMV-VSV-G was Addgene plasmid #8454 (deposited by Bob Weinberg), psPAX2 was Addgene plasmid #12260 (deposited by Didier Trono). pcDNA3-myc-RAB5-WT and -Q79L plasmids were a kind gift from Professor Harald Stenmark at the Institute for Cancer Research, Oslo University Hospital, Norway.

### Virus production and transduction

10^6^ HEK-FT cells were plated in a 10 cm dish and transfected next day with 1.6 µg of each of pCMV-VSV-G, psPAX2 and transfer plasmids. The medium was changed after 24 h and lentivirus-containing medium was collected and filtered through Acrodisc 0.45 µm Supor membrane syringe filter at 48 and 72 h after transfection. For cell infection, 10^5^ HeLa or U2OS cells were plated in wells of six-well plate and next day cell medium was exchanged for 2 ml fresh media with 0.5 ml of lentivirus-containing medium and 8 µg/ml of polybrene (Santa Cruz Biotechnology). Medium was changed 24 h after infection for fresh medium containing 2 µg/ml of puromycin. The pool of puromycin-selected cells was used for experiments.

### Generation of stably transfected cell lines

HeLa T-Rex FlpIN^WT^, ALFY^KO^ and U2OS cells were co-transfected with pOG44 Flp-recombinase expression vector (Invitrogen) and pDEST-FlpIN-EGFP-ALFY, LIR^mut^, FYVE^mut^, ΔPH-BEACH or ΔWD40-FYVE (for HeLa cells) or pDest-FlpIN-tdNGFlag-ALFY (for U2OS cells) in a ratio 10:1 using X-tremeGene9 DNA Transfection reagent (Roche, XTG9-RO). At 24 h post transfection, the cells were split into four 10 cm dishes andmedium containing 200 µg/ml Hygromycin B (VWR) was added to select for cells containing the gene of interest stably integrated into the genome. Individual clones were picked 2 weeks after selection and tested by western blotting and confocal imaging for EGFP–ALFY expression. mScarlet-I–RAB5A, mScarlet-I–RAB7A, mScarlet-I–RAB11A, mScarlet-I–LC3B, mScarlet-I–GABARAP, mScarlet-I–SQSTM1 and paxillin–mScarlet-I were generated by infection with lentiviral particles as described in the Virus production and transduction section. Lipofectamine 2000 (Invitrogen, 11668019) was used for transient transfections.

### Generation of the ALFY KO cell line by CRISPR/Cas9

To generate the ALFY KO cell line, CRISPR plasmids were made as described in the Zhang lab cloning protocol (Ran et al., 2013). Two independent single-guide RNAs (#1, 5′-GATCGGGAGCGTTTTAGAGG-3′; #2, 5′-GCAGAGTGAAGAAGCCAGTAG-3′) were designed using the crispr.mit.edu CRISPR design tool and obtained from Sigma (Fig. S1D). The guides were cloned into the hSpCas9-2A-Puro V2.0 (px459) plasmid (Addgene, #62988) and transfected into HeLa TRex FlpIN cells using X-tremeGene9 transfection reagent (Roche) according to the manufacturer's protocol. After 24 h, cells were treated with selection medium containing 2.0 μg/ml puromycin. After 3 days, puromycin-resistant cells were seeded as single cell per well density in a 96-well plate by serial dilution. Several clones were tested, and the knock-out clones were confirmed by western blotting.

### Cell lysis and western blotting

For western blot analysis, cells grown to confluency were harvested in lysis buffer (150 mM NaCl, 1% Triton X-100, 1 mM EDTA and 50 mM Tris-HCl pH 7.4) supplemented with complete protease inhibitor cocktail (Roche, 05056489001) for 10 min on ice. The lysates were centrifuged at 15,000 ***g*** for 10 min at 4°C to pellet cell debris. The protein concentration of supernatant was measured using the BCA protein assay (Pierce, 23225) to ensure loading of equal amounts on SDS-PAGE. Following SDS-PAGE, western blotting was performed using primary antibodies and fluorophore- or HRP-conjugated secondary antibodies for detection and analysis with the Chemidoc™ MP Imaging System (BioRad). Prior to detection, membranes containing HRP-conjugated antibodies were incubated with Supersignal™ West Dura Extended duration substrate (Thermo Fisher Scientific, 34075) for 5 min.

For western blots of EGFR, cells were serum starved for 2 h to induce expression of EGFR before treatment with 50 ng/ml EGF (Santa Cruz Biotechnology, sc-4552) for the indicated time points. The cells were subsequently lysed and prepared for western blotting as described above.

For treatment with PNGaseF and calf intestinal phosphatase (CIP), 3×10^5^ HeLa WT or ALFY KO^2-9^ cells were plated in wells of six-well plates and lysed in 100 µl/well of 50 mM Tris-HCl pH 7.5, 150 mM NaCl, 1% Triton X-100 the next day. Cell lysates were precleared by centrifugation at 12,000 ***g*** for 5 min. For treatment with PNGaseF, 9 µl of cell lysate was mixed with 1 µl of glycoproteins denaturing buffer (NEB, B1704S), heated to 100°C for 10 min, then cooled down on ice, supplemented with 2 µl of 10× GlycoBuffer2 (NEB, B3704S), 2 µl of 10% NP40, 1 µl of PNGaseF (NEB, P0709S) and 5 µl of water and incubated for 1 h at 37°C. For treatment with CIP, 9 µl of cell lysate was supplemented with 1.1 µl of 10× NEBuffer2 and 1 µl of CIP (NEB, M0290S) and incubated for 1 h at 37°C.

### Immunoprecipitation

For immunoprecipitation, cells were lysed in a buffer containing 150 mM NaCl, 1% Triton X-100 and 50 mM Tris-HCl pH 8.0 supplemented with complete protease inhibitor cocktail (Roche, 05056489001) (500 µl/10 cm dish). The lysates were incubated rotating at 4°C for 30 min. The lysates were centrifuged at 16,000 ***g*** for 10 min at 4°C, and the supernatants were incubated with 5 µl anti-ALFY antibodies rotating at 4°C overnight. Immunoprecipitation was performed by adding 20 µl Protein G Dynabeads (Invitrogen, 10003D) to the lysates for 1 h rotating at 4°C. The bound protein was collected using a DynaMag magnet (12321D) and washed three times in lysis buffer. The beads were washed twice with PBS and resuspended in 50 µl PBS before mass spectrometry analysis or western blot analysis.

### Transferrin recycling and flow cytometry analysis

To measure Tfn recycling, WT and ALFY KO cells were first placed on ice for 10 min, before treatment with 10 µg/ml Alexa Fluor 555-conjugated Tfn (Invitrogen, T35352) for 15 min. The cells were then washed with PBS and chased for the indicated time periods in cell culture conditions at 37°C. After chase, the cells were trypsinized, fixed with 4% paraformaldehyde (PFA) and centrifuged for 3 min at 500 ***g*** to pellet the cells. The cells were washed in PBS and separated into single cells by passing them through a cell strainer cap attached to a 5 ml tube (falcon, 352235). The cells were analyzed by flow cytometry using the NovoCyte flow cytometer from Acea Biosciences.

### Rhodamine–EGF degradation analysis

Cells grown in glass bottom eight-well chambers were treated with 50 ng/ml Rhodamine–EGF (Invitrogen, E348) for 15 min at 37°C, followed by either 60, 30 or 0 min chase, before fixation with 4% PFA (Polysciences, 18814-10) for 15 min on ice. 1× PBS was added to each well and images of Rhodamine–EGF were acquired using an Andor Dragonfly 505 spinning-disc confocal microscope using a NIKON Apo TIRF 60×/1.49 NA oil immersion objective. The whole-cell volume was imaged by acquiring a series of z-stacks with 0.3 µm axial distance. For image analysis of Rhodamine–EGF, *z*-stack maximum intensity projections were used to segment Rhodamine–EGF vesicles in cells. The average count and size (area) of Rhodamine–EGF-positive vesicles were quantified using CellProfiler (v. 4.1.3) (Carpenter et al., 2006).

### Immunofluorescence, confocal microscopy and colocalization analysis

Cells were grown on glass cover slips or in glass bottom 8-well chambers and treated as described before fixation in 4% paraformaldehyde (PFA; Polysciences, 18814-10) for 15 min at room temperature. The PFA was quenched in 0.05 M NH_4_Cl for 10 min followed by permeabilization for 5 min in PBS with 0.05% saponin. Immunostaining was performed by incubating the fixed cells with the indicated primary and corresponding secondary antibodies diluted in PBS with 0.05% saponin. The cells were subsequently stained for 10 min with 1 g/ml Hoechst 33342 diluted in PBS. Cover slips were mounted in Prolong Diamond Antifade Mountant (Invitrogen, p36965). The cells were analyzed using a Zeiss LSM 710 confocal microscope with a 63× objective lens.

Colocalization histograms were generated using ImageJ (Fiji), by drawing a line through the structure of interest and obtaining the grey values (intensity) per pixel using the plot profile function. The intensities of the two overlapping channels were plotted against each other for generating the graphs.

### Live cell imaging

Cells grown in eighth-well Lab-Tek II chambered coverglass were imaged live in FluoreBrite DMEM medium (Gibco) on Andor Dragonfly 505 high speed confocal platform equipped with Okolab cell incubator with temperature, CO_2_ and humidity control, using NIKON Apo TIRF 60×/1.49 NA oil immersion objective. The spinning disc confocal mode was used for all figures, except Fig. 1B, right panel, where TIRF mode with 100 nm penetration depth was used.

### Wound healing assay and directional analysis

Cells were seeded in 96-well ImageLock™ plates (Essen Bioscience). When the cells became a confluent monolayer, wounds were made using the WoundMaker tool (Essen Bioscience). To remove cell debris, the medium was changed, and the plate was subsequently placed in the IncuCyte^®^ S3 Live Cell microscope (Sartorius) for imaging. One image per well was captured every 10 min for 28 h to monitor cell migration into the wound. Relative wound density was quantified using the IncuCyte^®^ Software S3 (Sartorius), measuring the spatial cell density in the wound area relative to the spatial cell density outside of the wound area at every time point. The data is presented as the mean relative wound density using the mean of three replicate wells from three independent experiments.

Manual tracking of cells from the wound healing assay was performed using the ImageJ software with the Manual Tracking plugin and the Chemotaxis and Migration tool (ibidi GmbH). The persistence of cells was calculated by dividing the Euclidean distance by the accumulated distance obtained by the ibidi Chemotaxis and Migration tool.

For directional analysis of cells using immunofluorescence, cells were grown to confluency in two-well silicone inserts with a 500 µm cell free gap (ibidi). The inserts were removed, and the cells were incubated in fresh medium for 4–5 h, before fixation and staining against cortactin as mentioned above to mark the leading edge. For analysis by confocal imaging, 5–10 images were captured from three independent experiments. For quantification, cells with cortactin-positive leading edge were manually counted and compared to the total number of cells in each field of view, and a total of 60–100 cells from each condition were counted.

### Cell proliferation assay

Cell proliferation was measured with an 3-(4,5-dimethylthiazol-2-yl)-2,5-diphenyltetrazolium bromide (MTT) assay using a cell proliferation and cytotoxicity kit (Boster, AR1156) according to the manufacturer's instructions. Cells were seeded in triplicates at different cell densities in a 96-well plate. At 24 h after seeding, MTT was added to the medium and left for 4 h for the viable, proliferating cells to form purple insoluble formazan crystals. The crystals were solubilized overnight, and the concentration of the resulting colored solution was determined by measuring the optical density at 560 nm.

### Cell attachment assay

The different cell lines were trypsinized and stained with CellMask™ Deep Red Plasma membrane stain (Invitrogen, C10046) before subsequent seeding in a 96-well plate (six replicates per cell line). The cells were incubated for 30 min to allow cells to attach to the well bottom before three washes in PBS to remove the non-attached cells. The 96-well plate was scanned using Odyssey CLx Imager using the 700 laser and total intensity per well was quantified using the Image Studio™ Software as a measure of the remaining cells.

### Statistical analysis

Significance was determined using GraphPad Prism 8.0.1 and *P*-values were derived from two-tailed *t*-tests for unpaired samples and considered statistically significant at *P*≤0.05 (see figure legends for further details).

## Supplementary Material

Supplementary information

Reviewer comments
