# Supplementary material for: ALFY localizes to early endosomes and cellular protrusions to facilitate directional cell migration
Source: J Cell Sci. 2022 Feb 18;135(4):jcs259138. doi: 10.1242/jcs.259138 (PMC8919339; doi:10.1242/jcs.259138)
Supplement: Supplementary information [file joces-135-259138-s1.pdf]

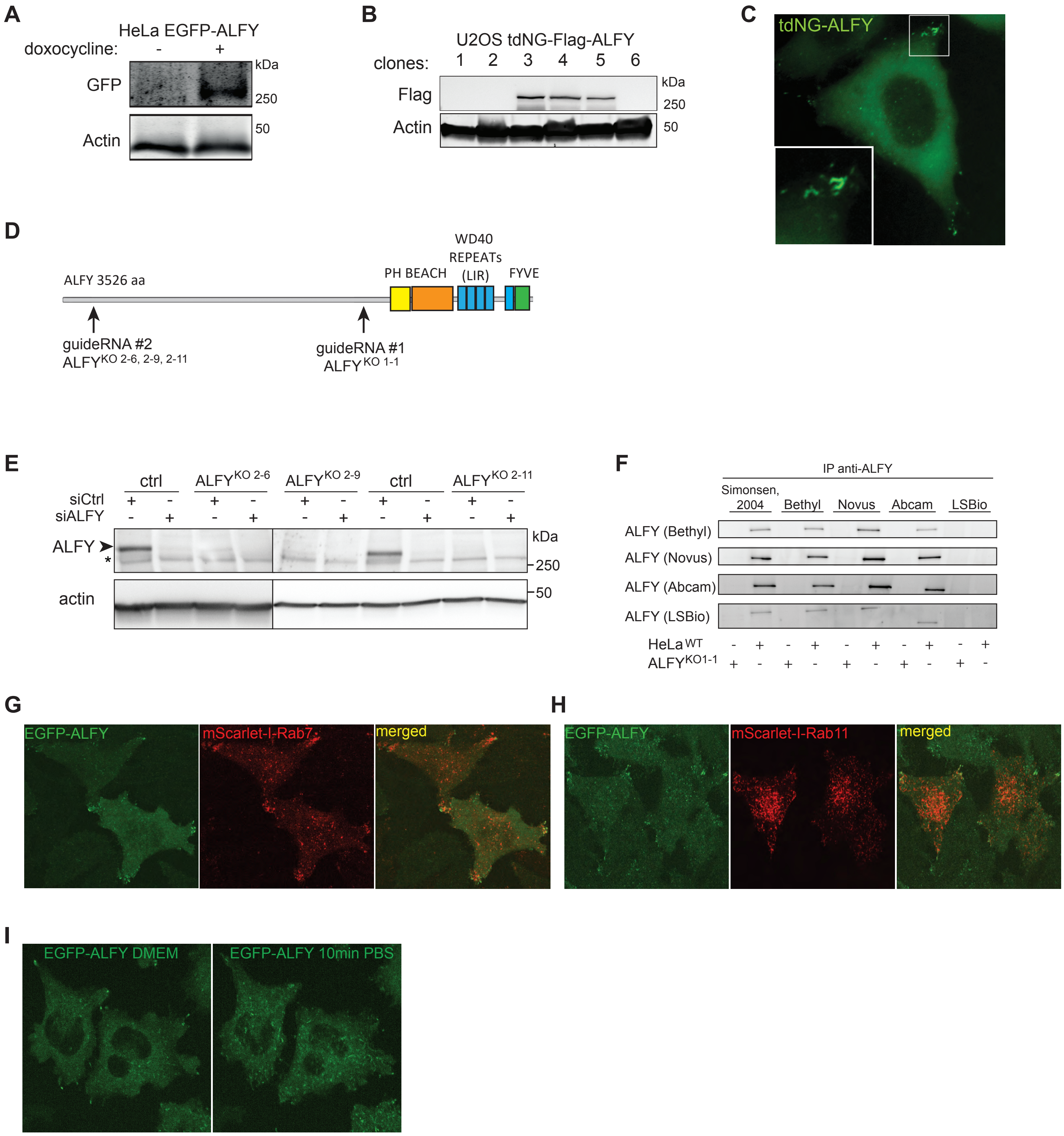

Journal of Cell Science • Supplementary information

Fig. S1. A. HeLa cells stably transfected with EGFP-ALFY were treated with doxocycline for 24 hours to induce expression of EGFP-ALFY, followed by western blot analysis. **B.** Western blot analysis of different clones of U2OS cells stably expressing full length ALFY fused to a tag containing a tandem-dimer of NeonGreen with 3xFlag as a linker (tdNGFlag).

C. Representative image of U2OS cells stably expressing the tdNGFlag-ALFY. **D.** Two CRISPR guides targeting ALFY were used to generate the ALFY KO cell lines. One guide targeting exon 6 (coding aa 102-138) (generating ALFY$^{KO\ 2\text{-}6}$, ALFY$^{KO\ 2\text{-}9}$ and ALFY$^{KO\ 2\text{-}11}$) and one guide targeting exon 47 (coding aa 2481-2535) (generating ALFY$^{KO\ 1\text{-}1}$).

E. Western blot analysis of endogenous ALFY protein levels to confirm KO of the ALFY$^{KO\ 2\text{-}6}$, ALFY$^{KO\ 2\text{-}9}$ and ALFY$^{KO\ 2\text{-}11}$ cell lines. To verify the correct ALFY band the cells were treated with either control siRNA or siRNA against ALFY. **F.** Immunoprecipitation of endogenous ALFY from HeLa T-Rex FlpIn^{WT} and ALFY$^{KO\ 1\text{-}1}$ cells was performed using different antibodies recognizing either the N- or C-terminus of ALFY, followed by western blotting with the same antibodies. **G.** and **H.** ALFY$^{KO1\text{-}1}$-EGFP-ALFY cellsstably expressing mScarlet-I-Rab7A (**G.**) or mScarlet-I-Rab11A (**H.**) were treated with tetracycline overnight and analysed with live cell imaging. **I.** ALFY$^{KO1\text{-}1}$-EGFP-ALFY cells, treated overnight with tetracycline and imaged live in DMEM, 37°C (left) or 10 min after exchange of media for cold PBS (right).

Journal of Cell Science • Supplementary information

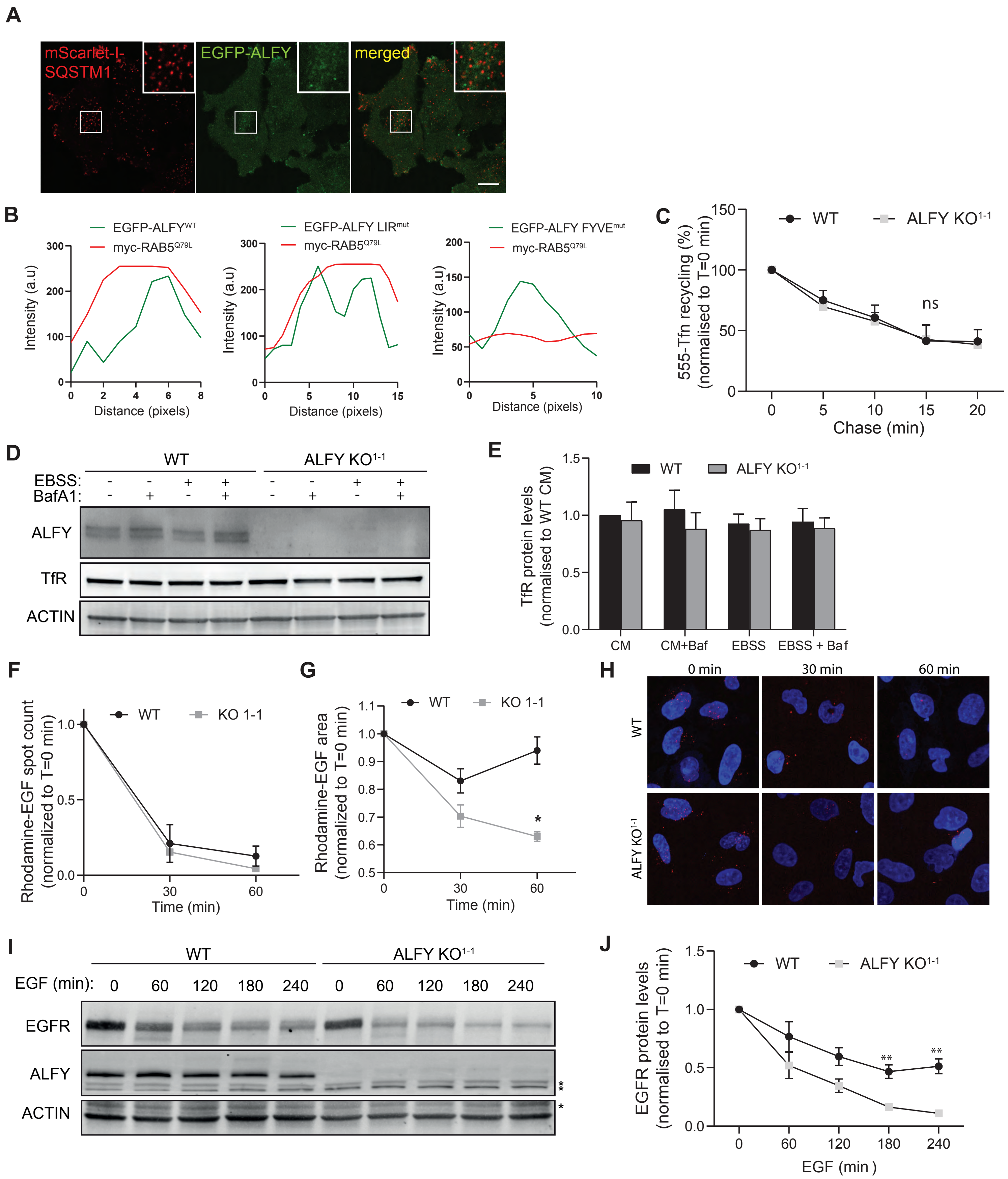

Journal of Cell Science • Supplementary information

Fig. S2. A. ALFY^{KO1-1}-EGFP-ALFY cells stably expressing mScarlet-I-SQSTM1 were treated with tetracycline overnight and analyzed with live cell imaging. Scale bar = 10 µm. **B**. Colocalization histograms of EGFP-ALFY^{WT} and myc-RAB5^{Q79L}, generated from the EGFP-ALFY vesicles in **Fig. 2C** marked with a small arrow. **C**. 555-transferrin recycling in WT and ALFY$^{KO\ 1-1}$ analyzed by flow cytometry (n=3 for T=0 and 15 min, n=2 for T=5, 10 and 20 min) **D**. Western blot analysis of transferrin receptor protein levels in WT and ALFY$^{KO\ 1-1}$ cells starved or not in the prescence or absence of the lysosomal inhibitor BafA1.

E. Quantification of the TfR protein bands in **D** (n=3, $p<0.05$ by student's t-test)**. F**. WT and ALFY^{KO1-1} cells were treated with Rhodamine-EGF and chased for the indicated time points before fixation and imaging. Graph shows number of Rh-EGF spots (mean values of n=3 independent experiments).

G. Quantification of Rh-EGF area (mean values of n=3 independent experiments) **H.** Representative images of the cells treated with Rhodamine-EGF (quantified in **F** and **G**). **I.** WT and ALFY^{KO1-1} cells were treated with EGF for the indicated time points before western blot analysis of the EGFR protein levels.

J. The EGFR protein bands from **I.** were quantified (n=3, $p<0.05$ by student's t-test).

Journal of Cell Science • Supplementary information

J. Cell Sci.: doi:10.1242/jcs.259138: Supplementary information

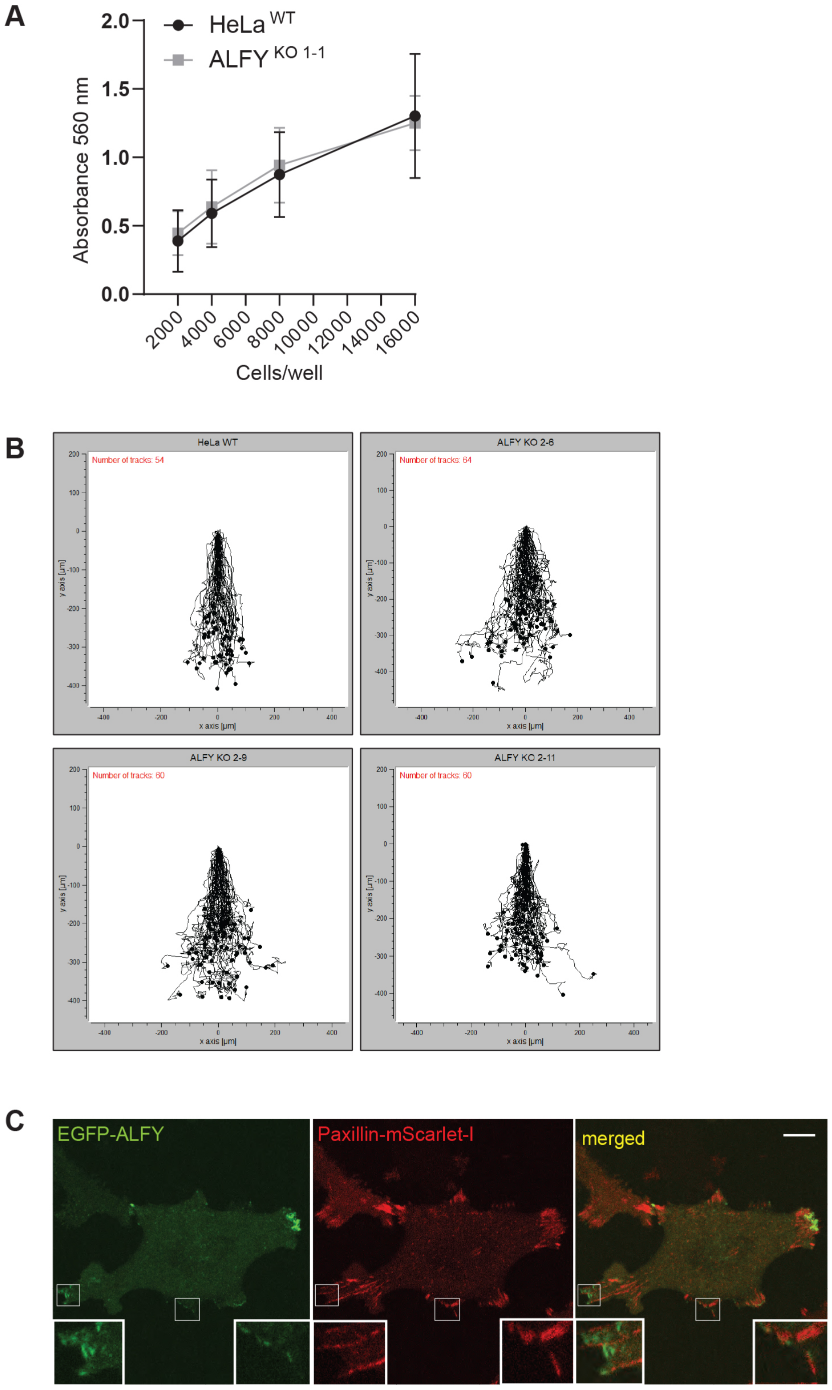

Fig. S3. A. HeLa^{WT} and ALFY$^{KO\ 1\text{-}1}$ cells were seeded at the indicated cell densities and cell proliferation was measured after 24 hours using the MTT-assay, measuring the absorbance of solubilized formazan crystals at 560 nm. (n=4, $p<0.05$ by student's t-test). **B.** Graphs representing the movement of HeLa^{WT}, ALFY$^{KO\ 2\text{-}6}$, ALFY$^{KO\ 2\text{-}9}$ and ALFY$^{KO\ 2\text{-}11}$ cells obtained by tracking cells from three individual wound healing experiments, using the manual tracking plugin of ImageJ and the Chemotaxis and migration tool (Ibidi). **C.** ALFY$^{KO\ 1\text{-}1}$ EGFP-ALFY cells stably expressing paxillin-mScarlet-I were treated with tetracycline overnight and analyzed with live cell imaging. Scale bar = 10 µm.

Journal of Cell Science • Supplementary information

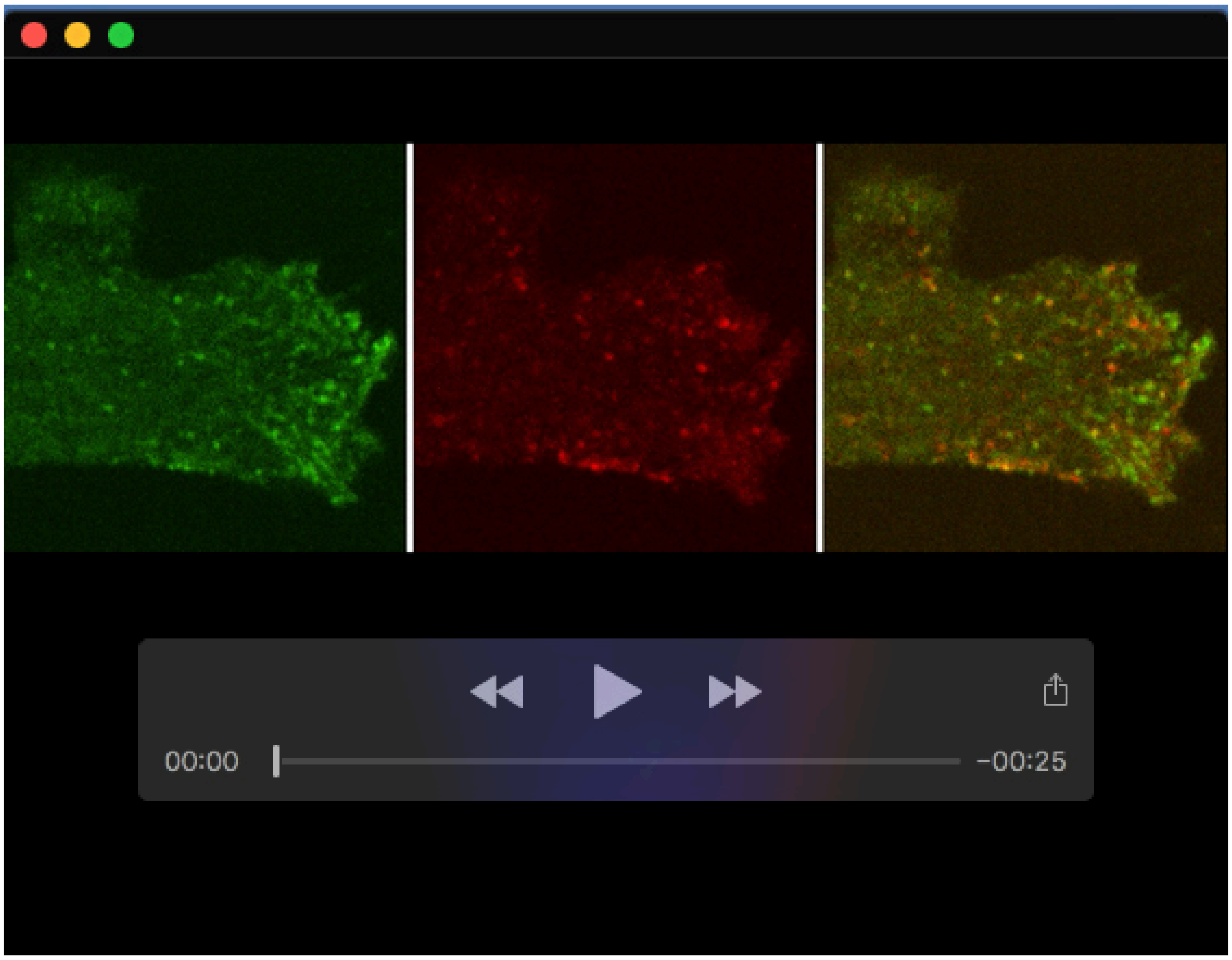

Movie 1. Live cell imaging of HeLa cells stably transfected with EGFP-ALFY and mScarlet-I-RAB5A

Journal of Cell Science • Supplementary information

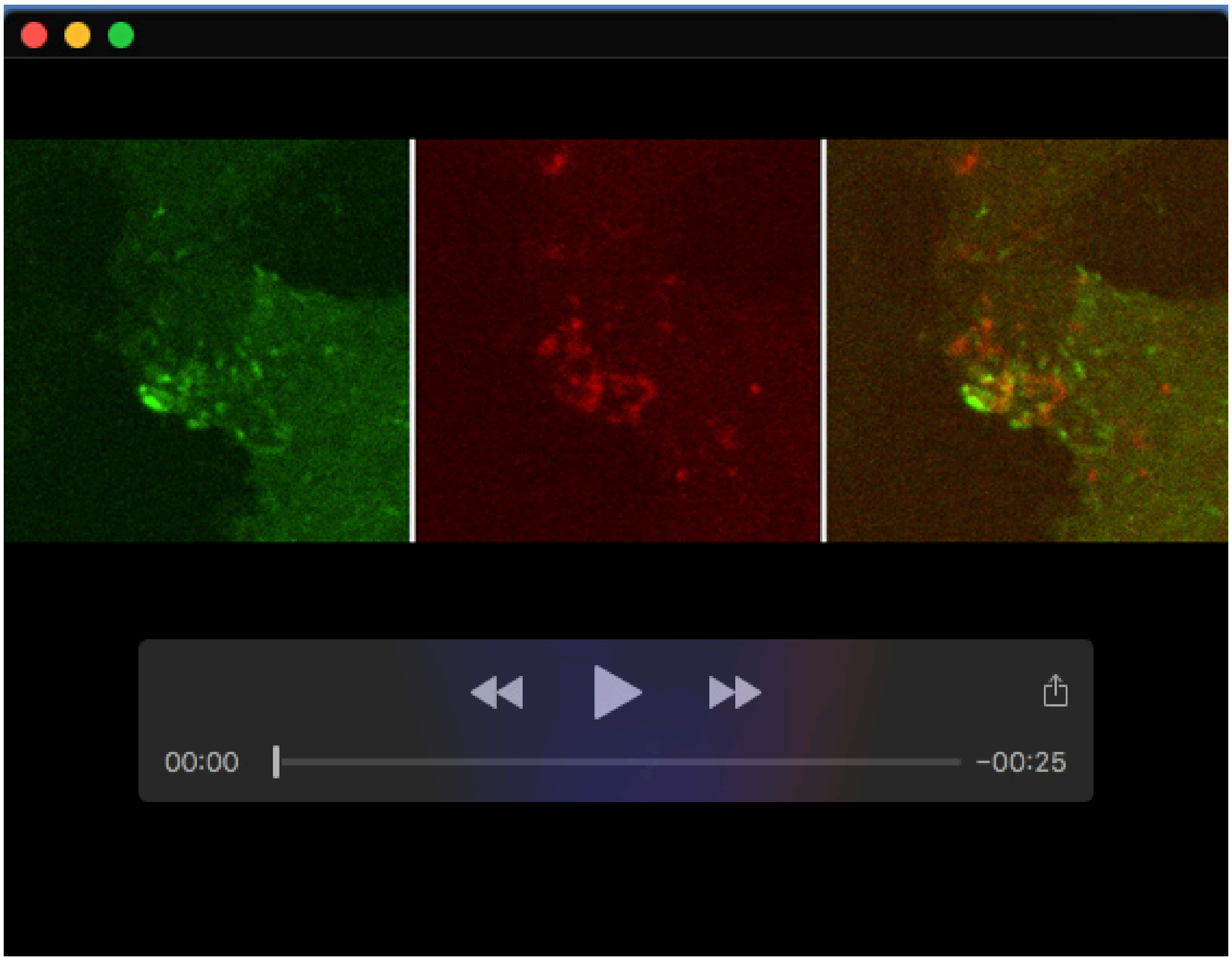

Movie 2. Live cell imaging of HeLa cells stably transfected with EGFP-ALFY and mScarlet-I-RAB7A

Journal of Cell Science • Supplementary information

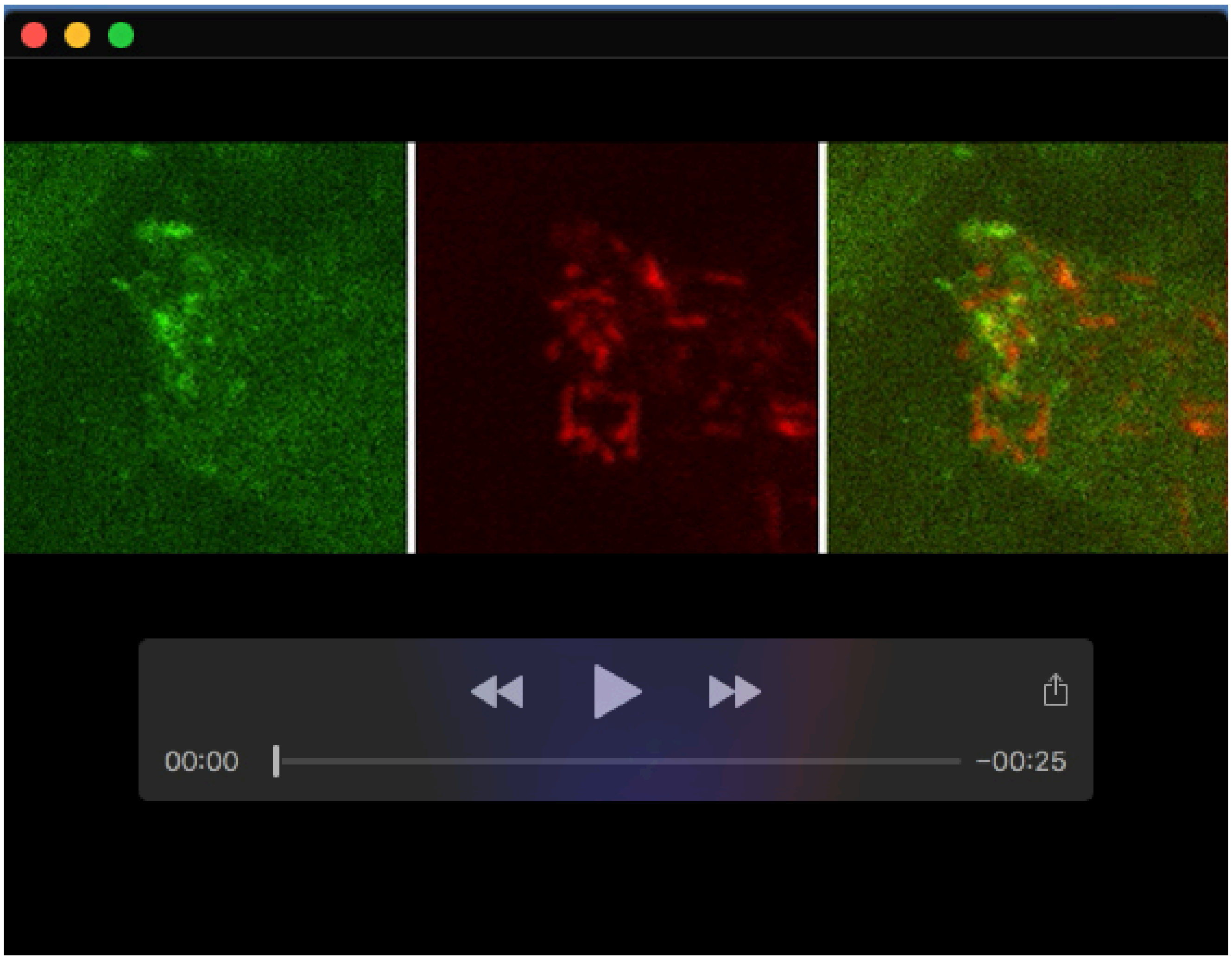

Movie 3. Live cell imaging of HeLa cells stably transfected with EGFP-ALFY and mScarlet-I-RAB11A

Journal of Cell Science • Supplementary information
