# Supplementary material for: ALFY localizes to early endosomes and cellular protrusions to facilitate directional cell migration
Source: J Cell Sci. 2022 Feb 18;135(4):jcs259138. doi: 10.1242/jcs.259138 (PMC8919339; doi:10.1242/jcs.259138)
Supplement: Reviewer comments [file joces-135-259138_review_history.pdf]

ALFY localizes to early endosomes and cellular protrusions to facilitate directional cell migration

Kristiane Søreng, Serhiy Pankiv, Camilla Bergsmark, Ellen M. Haugsten, Anette K. Dahl, Laura R. de la Ballina, Ai Yamamoto, Alf H. Lystad and Anne Simonsen
DOI: 10.1242/jcs.259138

Editor: Tamotsu Yoshimori

Review timeline

Original submission:	15 July 2021
Editorial decision:	18 August 2021
First revision received:	13 December 2021
Accepted:	14 January 2022

Original submission

First decision letter

MS ID#: JOCES/2021/259138

MS TITLE: ALFY localizes to early endosomes and cellular protrusions to facilitate directional cell migration

AUTHORS: Kristiane Søreng, Serhiy Pankiv, Camilla Bergsmark, Ellen M Haugsten, Ai Yamamoto, Alf H Lystad, and Anne Simonsen
ARTICLE TYPE: Short Report

We have now reached a decision on the above manuscript.

To see the reviewers' reports and a copy of this decision letter, please go to: https://submit-jcs.biologists.org and click on the 'Manuscripts with Decisions' queue in the Author Area. (Corresponding author only has access to reviews.)

As you will see, the reviewers raise a number of substantial criticisms that prevent me from accepting the paper at this stage. They suggest, however, that a revised version might prove acceptable, if you can address their concerns. If you think that you can deal satisfactorily with the criticisms on revision, I would be pleased to see a revised manuscript. We would then return it to the reviewers.

We are aware that you may be experiencing disruption to the normal running of your lab that makes experimental revisions challenging. If it would be helpful, we encourage you to contact us to discuss your revision in greater detail. Please send us a point-by-point response indicating where you are able to address concerns raised (either experimentally or by changes to the text) and where you will not be able to do so within the normal timeframe of a revision. We will then provide further guidance. Please also note that we are happy to extend revision timeframes as necessary.

Please ensure that you clearly highlight all changes made in the revised manuscript. Please avoid using 'Tracked changes' in Word files as these are lost in PDF conversion.

I should be grateful if you would also provide a point-by-point response detailing how you have dealt with the points raised by the reviewers in the 'Response to Reviewers' box. Please attend to

© 2022. Published by The Company of Biologists under the terms of the Creative Commons Attribution License (https://creativecommons.org/licenses/by/4.0/).

all of the reviewers' comments. If you do not agree with any of their criticisms or suggestions please explain clearly why this is so.

Reviewer 1

Advance summary and potential significance to field

This study identifies a novel molecule (ALFY) that is involved in the cellular motility. Their findings may explain the defect of neuronal migration and pathfinding phenotypes observed in mice lacking ALFY.

Comments for the author

In this report, the authors characterized an autophagy adaptor protein ALFY and found that ALFY localized at early endosomes, protrusions, and other vesicular structures. The authors suggested that ALFY is involved in cellular motility, possibly through its regulation on integrin trafficking. The results are potentially interesting, but several experiments are underdeveloped.
(1) The authors characterized the localization of two mutants (LIR-mut and FYVE-mut). Other mutants that lack PH domain or BEACH domain, should be prepared and examined. Then, the authors may be able to address which domain(s) of ALFY is required for its localization to protrusions and/or for the cellular motility.
(2) The images in Figure 1F and Figure 2 need quantitation. How about the co-localization with Rab11?
(3) In Figure 4B, This reviewer is concerned about why the band pattern of integrins is so different between “1-1” vs “the other ALFY-KO clones”.
(4) The authors should perform some sort of integrin “trafficking” assay. No direct data were provided in Figure 4.
(5) Because the authors found that some ALFY localized at early endosomes, they should assay the rate of EGF degradation and that of transferrin recycling, which are common routine for validating the endocytic traffic.
Minor critique:
(6) “doxocycline” should read “doxycycline”.

Reviewer 2

Advance summary and potential significance to field

This paper by Soreng et al. reveals subcellular localization mechanisms of an endosomal protein ALFY. By establishing several ALFY knockout cells, the authors further demonstrate that in the absence of ALFY, cells migrate faster but less directionally, causing impaired closure in the wound scratch assay. In ALFY knockout cells, mobility of several integrin molecules changed in SDS-PAGE providing evidence of causative mechanisms for the altered cell migration.
Together, this paper nicely illustrates the role of ALFY-mediated endocytic pathways in the regulation of integrins and cell migration.

Comments for the author

Several issues need to be clarified to further strengthen the findings in this paper.
1) In p5, lines 1-8, the authors attribute the failure of early endosome localization of ALFY (in the previous study?) to the use of cold PBS before fixation. However, citation of the previous paper(s) is lacking. In which study was ALFY localization reported? How was it described? What protocol was employed? These must be introduced in more detail.

2) In addition, it is recommended to show the evidence of the effect of cold PBS on the ALFY localization. This can be examined either using their ALFY antibodies or observing EGFP-ALFY in live cells.

3) In p4 (bottom), the authors concluded that EGFP-ALFY colocalized exclusively with EEA1 and Rab5 but not with Rab7. I am not convinced with this in the Figure 2A-C images. Colocalization

© 2022. Published by The Company of Biologists under the terms of the Creative Commons Attribution License (https://creativecommons.org/licenses/by/4.0/).

appear (to me) not so ‘exclusive’ with EEA1 and Rab5. Rab7 is showing some limited overlapping distribution with EGFP-ALFY.

Please clarify these issues.
4) The data in Figure 4B is a very important finding in this paper. The band shift, especially that of Integrin beta1, appears to arise from altered glycosylation. Because N-glycosylation of alpha5 and beta 1 integrins is important for cell migration (JBC 284, 12207-216, 2009; MCB 37, e00558-16 2017), it is highly suggested to examine which of glycosylation or phosphorylation is responsible for the band shift using deglycosylation enzymes and phosphatases. Also add some discussion on the possible link between recycling and modification of integrins.
5) In p6 (middle~bottom), protein levels of alphaV, beta3 and paxillin are described only for ALFY-KO1-1 cells. This is confusing because beta3 and paxillin changed differently in other KO cells. Clarify this by adding findings in other KO cell lines.
6) Figure S1 legend, line1: “tetracycline” to “doxycycline” Correct “toinduce”.

First revision

Author response to reviewers' comments

We would like to thank the reviewers for their encouraging and helpful comments. We have addressed all concerns and points raised by the reviewers. Please find a detailed point-by-point response to each of the reviewer’s point below.

Reviewer 1

Advance Summary and Potential Significance to Field:
This study identifies a novel molecule (ALFY) that is involved in the cellular motility. Their findings may explain the defect of neuronal migration and pathfinding phenotypes observed in mice lacking ALFY.

Reviewer 1 Comments for the Author:
In this report, the authors characterized an autophagy adaptor protein ALFY and found that ALFY localized at early endosomes, protrusions, and other vesicular structures. The authors suggested that ALFY is involved in cellular motility, possibly through its regulation on integrin trafficking. The results are potentially interesting, but several experiments are underdeveloped.

(1) The authors characterized the localization of two mutants (LIR-mut and FYVE-mut). Other mutants that lack PH domain or BEACH domain, should be prepared and examined. Then, the authors may be able to address which domain(s) of ALFY is required for its localization to protrusions and/or for the cellular motility.

Response: We agree that it would be important to identify the region(s) of ALFY needed for its localization to protrusions in order to understand the mechanisms underlying its role in cellular motility. As suggested by the reviewer, we have now generated stable cell lines expressing EGFP-ALFY lacking the PH-BEACH domains (ΔPH-BEACH) or the WD40-FYVE domains (ΔWD40-FYVE) (new Fig. 1F- G). The localization of ALFY to protrusions is lost in both cases, demonstrating that both the PH-BEACH and the WD40-FYVE region, the latter containing the LIR and FYVE, are important for ALFY’s localization to cell protrusions. It is also interesting to note that EGFP-ALFY remains bound to intracellular vesicles in both these cell lines, indicating a role for the N-terminus in recruitment to vesicles.

(2) The images in Figure 1F and Figure 2 need quantitation. How about the co-localization with Rab11?

Response: We have now quantified the images in Figure 1F as the percentage of cells having ALFY positive protrusions, showing that approx. 80% of the cells expressing EGFP-ALFY demonstrate

© 2022. Published by The Company of Biologists under the terms of the Creative Commons Attribution License (https://creativecommons.org/licenses/by/4.0/).

localization of ALFY to cellular protrusion (new Fig. 1G). There is no significant differences in cells treated with the VPS34 inhibitor IN1 or in cells expressing ALFY LIR or FYVE mutant, but the localization of ALFY to protrusions is generally lost in cells expressing ΔPH-BEACH or ΔWD40-FYVE (new Fig. 1F-G).

To quantify the data in Figure 2, showing colocalization (or not) of EGFP-ALFY with different cellular markers (EEA1, mScralet-RAB5, myc-RAB5Q79L, mScralet-LC3B or mScralet-GABARAP) we have now included fluorescent intensity histograms of the indicated EGFP-ALFY positive structures (new Fig. 2A- E).

We have now generated stable cell lines expressing EGFP-ALFY and mScarlet-RAB11. We cannot detect any colocalization between the two proteins in fixed cells, but do observe transient "kiss-and-run" interactions by live imaging (video attached). We now also include videos of cells expressing EGFP- ALFY with mScarlet-RAB5 and mScarlet-RAB7 (in response to comment 3 from reviewer #2). ALFY clearly move together with RAB5 positive vesicles. However, although we see both ALFY and RAB7 in protrusions, they either localize to different vesicles or interact in a kiss-and-run dependent manner.

(3) In Figure 4B, This reviewer is concerned about why the band pattern of integrins is so different between "1-1" vs "the other ALFY-KO clones".

Response: We are not so concerned about this difference, but rather find it quite interesting and also reassuring to see that all ALFY KO clones affect intergrin levels (using two different guide RNAs) and that we can rescue this by expression of full length ALFY. The KO 1-1 clone is generated using gRNA targeting the PH-BEACH region of ALFY, which potentially can allow expression of N-terminal parts of ALFY (although we do not detect any truncated proteins with the available antibodies, recognizing both N- and C-terminal part of ALFY, Fig. S1F). The other KO clones (2-6, 2-9, 2-11) were generated using a gRNA targeting the N-terminal part of ALFY and again no extra bands were detected. ALFY is a very large protein (3526 amino acids with 68 exons) and several transcripts have been detected. Thus, we cannot rule out that some smaller parts of ALFY are expressed in these cells that potentially could have dominant negative functions.

In response to reviewer #2 (comment 4) we have now further investigated this phenotype by adding PNGaseF enzyme to cleave N-glycans or calf intenstinal phosphatase (CIP) to remove phosphate. The mobility shift difference of Integrin-α5 and Integrin-β1 seen in ALFY KO cells was completely lost in cells treated with PNGaseF, while CIP treatment had no effect (new Fig. 4C), demonstrating that this mobility shift is due to altered N-glycosylation. It is known that both Integrin α5 and integrin β1 contain several N-linked glycosylation sites and that, depending on the glycans added, affect the heterodimerization and binding properties of α5β1 integrin, as well as cell migration and adhesion (Isaji et al., 2009, Isaji et al., 2006, Marsico et al., 2018, Hang et al., 2017). We do observe a colocalization of EGFP-ALFY with Integrin-α5-mScarlet-I in cell protrusions and in intracellular structures (Fig. 4D), but believe that a deeper understanding of how ALFY regulates glycosylation and/or trafficking of integrins is beyond the scope of this manuscript.

(4) The authors should perform some sort of integrin "trafficking" assay. No direct data were provided in Figure 4.

Response: We agree that it would be important to show a functional effect on integrin trafficking in cells depleted of ALFY and we have indeed tried various experimental approaches to address this;

1) Wild type and ALFY KO cells were subjected to surface biotinylation, followed by internalization at different time points (with de-biotinylation of surface bound proteins), addition of cell lysate to a plate coated with anti-Integrin α5 antibody, wash and finally incubation with streptavidin-HRP, followed by a colorimetric substrate for HRP. Unfortunetely, this assay did not work, most likely due to inefficient de-biotinylation of surface-bound proteins (our baseline control).
2) Colocalization of Integrin α5 with EEA1 and RAB11 (endogenous antibodies) in WT and ALFY KO cells. We could not detect any difference in the colocalization of Integrin α5 with EEA1 (data not shown). Unfortunately, neither of the anti-RAB11 antibodies available to us showed specific staining and we were therefore not able to conclude about a possible difference in colocalization

© 2022. Published by The Company of Biologists under the terms of the Creative Commons Attribution License (https://creativecommons.org/licenses/by/4.0/).

with RAB11.

As mentioned above, we now demonstrate that ALFY regulate glycosylation of integrin α5 and integrin β1 (new Fig. 4C). Integrins can be glycosylated in RAB11 positive recycling endosomes (Kitano et al., 2021) and trans-Golgi network or perinuclear recycling compartments, in which integrins transverse during long-loop recycling (De Franceschi et al., 2015). Thus, the localization of ALFY to early endosomes and its observed close apposition and short-term co-localization with RAB11A and RAB7A (Fig. S1G and S1H, Movie 2 and 3) might explain the observed difference in integrin α5β1 glycosylation pattern in ALFY KO cells. It is also interesting to note that the N-glycosylation of integrin α5 has been reported to regulate EGFR activation (Hang et al., 2015), which may explain the observed effect of ALFY KO on EGFR turnover (new Fig. S2 E-J).

(5) Because the authors found that some ALFY localized at early endosomes, they should assay the rate of EGF degradation and that of transferrin recycling, which are common routine for validating the endocytic traffic.

Response: We thank the reviewer for this suggestion. We have now included data showing that recycling of transferrin (555-Tfn) and protein levels of TfR are unaffected by ALFY KO (new Fig. S2C-E). In contrast, the EGF-induced turnover of EGFR and Rhodamine-EGF were significantly increased in ALFY KO cells compared to WT cells, indicating increased lysosomal transport (new Fig. S2F-J).

Minor critique:

(6) “doxocycline” should read “doxycycline”.

Response: Thanks - this has now been corrected.

Reviewer 2

Advance Summary and Potential Significance to Field:

This paper by Soreng et al. reveals subcellular localization mechanisms of an endosomal protein ALFY. By establishing several ALFY knockout cells, the authors further demonstrate that in the absence of ALFY, cells migrate faster but less directionally, causing impaired closure in the wound scratch assay. In ALFY knockout cells, mobility of several integrin molecules changed in SDS-PAGE, providing evidence of causative mechanisms for the altered cell migration. Together, this paper nicely illustrates the role of ALFY-mediated endocytic pathways in the regulation of integrins and cell migration.

Reviewer 2 Comments for the Author:
Several issues need to be clarified to further strengthen the findings in this paper.

1) In p5, lines 1-8, the authors attribute the failure of early endosome localization of ALFY (in the previous study?) to the use of cold PBS before fixation. However, citation of the previous paper(s) is lacking. In which study was ALFY localization reported? How was it described? What protocol was employed? These must be introduced in more detail.

Response: We apologize for not describing and citing the relevant previous papers and for the possible confusion. The fact that we previously have been unable to detect ALFY on early endosomes is not due to the use of cold PBS, but rather due to our failure to clone and express full length ALFY (which is a large protein of 3526 amino acids). Thus, we have relied on a rather unspecific anti-ALFY antibody and expression of ALFY deletion mutants, which do not show staining to early endosomes (most likely due to improper folding). We have now included the following sentence (page 4): “The large size of ALFY has made it difficult to express the full-length protein and previous studies have therefore been based on immunostaining of fixed cells with an anti-ALFY antibody or expression of deletion mutants (Clausen et al., 2010, Simonsen et al., 2004).”

© 2022. Published by The Company of Biologists under the terms of the Creative Commons Attribution License (https://creativecommons.org/licenses/by/4.0/).

The cold PBS does however alter the cellular localization of ALFY, as we no longer observe localization of ALFY to cellular protrusions upon addition of cold PBS, as observed by live imaging (see point below).

2) In addition, it is recommended to show the evidence of the effect of cold PBS on the ALFY localization. This can be examined either using their ALFY antibodies or observing EGFP-ALFY in live cells.

Response: We have now included images of EGFP-ALFY cells untreated and 10 min after treatment with cold PBS, analyzed by live imaging, showing redistribution of ALFY labelled vesicles from cell protrusions to more proximal cytosolic localization (new Suppl Fig. 1I).

3) In p4 (bottom), the authors concluded that EGFP-ALFY colocalized exclusively with EEA1 and Rab5 but not with Rab7. I am not convinced with this in the Figure 2A-C images. Colocalization appear (to me) not so ‘exclusive’ with EEA1 and Rab5. Rab7 is showing some limited overlapping distribution with EGFP-ALFY. Please clarify these issues.

Response: We agree that the original image showing EGFP-ALFY colocalization with mScarlet-RAB7 was unclear. We have now removed this and included a new image of cells expressing EGFP-ALFY and mScarlet-RAB7, as well as images and videos of cells with stable expression of EGFP-ALFY and mScarlet--RAB11 (new Fig. S1G-H and Movies 1-3). While we cannot detect any colocalization/co-migration between EGFP-ALFY and mScarlet-RAB7 or mScarlet-RAB11 positive vesicles (as we see for mScarlet- RAB5), we do observe transient dynamic “kiss-and-run” interactions by live imaging.

4) The data in Figure 4B is a very important finding in this paper. The band shift, especially that of Integrin beta1, appears to arise from altered glycosylation. Because N-glycosylation of alpha5 and beta 1 integrins is important for cell migration (JBC 284, 12207-216, 2009; MCB 37, e00558-16,2017), it is highly suggested to examine which of glycosylation or phosphorylation is responsible for the band shift using deglycosylation enzymes and phosphatases. Also add some discussion on the possible link between recycling and modification of integrins.

Response: We thank the reviewer for this comment. We have performed the suggested experiment, adding PNGaseF enzyme to cleave N-glycans or calf intenstinal phosphatase (CIP) to remove phosphate. This shows a complete loss of the mobility shift of Integrin-α5 and Integrin-β1 seen in ALFY KO cells when treated with PNGaseF, while CIP treatment had no effect (new Fig. 4C), demonstrating that this mobility shift is due to altered N-glycosylation of Integrin-α5 and Integrin-β1 in ALFY KO cells. We have now included the following text to the discussion of these findings (page 7) where we have cited the recommended papers:
“Both Integrin-α5 and Integrin-β1 contain several potential N-linked glycosylation sites, and their glycosylation can affect the heterodimerization and binding properties, cell migration and adhesion (Isaji et al., 2009, Isaji et al., 2006, Marsico et al., 2018, Hang et al., 2017). The mechanism of how ALFY regulates glycosylation of integrins is unclear, but its colocalization with Integrin- 5-mScarlet-I in cell protrusions and in intracellular structures (Fig. 4D), as well as with RAB5A and EEA1 (Fig 2A-B, Movie 1), suggest that it might regulate endocytosis of integrins from the plasma membrane and their transport to the recycling compartments. We observed close apposition and short-term co-localization of ALFY with the recycling endosome marker RAB11A and late endosome marker RAB7A (Fig. S1G-H, Movie 2-3), supporting this hypothesis. Glycosylation of integrins in RAB11 positive recycling endosomes (Kitano et al., 2021) and trans-Golgi network or perinuclear recycling compartments, where integrins transverse during long-loop recycling (De Franceschi et al., 2015), can explain the different glycosylation pattern of integrin-α5β1 in ALFY^{KO} cells. Interestingly, N-glycosylation of Integrin-α5 has been reported to regulate EGFR activation (Hang et al., 2015), which may explain the effect of ALFY^{KO} on EGFR turnover (Fig. S2F-J).»

5) In p6 (middle~bottom), protein levels of alphaV, beta3 and paxillin are described only for ALFY-KO1- 1 cells. This is confusing because beta3 and paxillin changed differently in other KO cells. Clarify this by adding findings in other KO cell lines.

© 2022. Published by The Company of Biologists under the terms of the Creative Commons Attribution License (https://creativecommons.org/licenses/by/4.0/).

Response: We apologize for this confusion. Fig. 4B shows western blot analysis of different integrins both for both ALFY KO clones (KO^{1-1} and $KO^{2-6,\ 2-9,\ 2-11}$ with rescue). We have now clarified and discussed the difference between the KOs in the text (page 6): “It is not clear why the two $ALFY^{KO}$ clones affect different integrins, but as ALFY is a very large protein with several transcripts, we cannot rule out that the two gRNAs (targeting different exons) result in expression of a part of ALFY that potentially could have dominant negative functions, although no truncated proteins were observed with the available antibodies (Fig. S1E-F). However, the changes in integrins observed upon ALFY-depletion could be rescued by expression of full-length ALFY, indicating that ALFY regulates trafficking and possibly post- translational modifications of integrins.”

6) Figure S1 legend, line1: “tetracycline” to “doxycycline” Correct “toinduce”.

Response: Thank you - these have now been corrected.

Second decision letter

MS ID#: JOCES/2021/259138

MS TITLE: ALFY localizes to early endosomes and cellular protrusions to facilitate directional cell migration

AUTHORS: Kristiane Søreng, Serhiy Pankiv, Camilla Bergsmark, Ellen M Haugsten, Anette K Dahl, Laura R de la Ballina, Ai Yamamoto, Alf H Lystad, and Anne Simonsen
ARTICLE TYPE: Short Report

I am happy to tell you that your manuscript has been accepted for publication in Journal of Cell Science, pending standard ethics checks.

Reviewer 1

Advance summary and potential significance to field

This study identifies a novel molecule (ALFY) that is involved in the cellular motility. Their findings may explain the defect of neuronal migration and pathfinding phenotypes observed in mice lacking ALFY.

Comments for the author

The authors adequately addressed to my original concerns. The new data in Fig .4C is neat, illustrating that ALFY is involved in the carbohydate modification of integrins, which is well known to be sensitive to integrin trafficking.
I believe that the Ms has been much improved by the revision and is ready for publication.

Reviewer 2

Advance summary and potential significance to field

The manuscript has been substantially improved.

Comments for the author

I have no further suggestion except that there still remains a typo
“doxocycline” in Figure 1E, Figure S1A and its legend.

© 2022. Published by The Company of Biologists under the terms of the Creative Commons Attribution License (https://creativecommons.org/licenses/by/4.0/).
